# Vertex: A Semantic Graph-Based Indoor Navigation System with Vision-Language Landmark Verification

**DOI:** 10.3390/s26072031

**Published:** 2026-03-24

**Authors:** Isabel Ferri-Molla, Dena Bazazian, Marius N. Varga, Jordi Linares-Pellicer, Joan Albert Silvestre-Cerdà

**Affiliations:** 1 Valencian Research Institute for Artificial Intelligence (VRAIN), Universitat Politècnica de València (UPV), Camí de Vera, s/n, 46022 Valencia, Spain; isfermol@upv.es (I.F.-M.); jlinares@dsic.upv.es (J.L.-P.); 2 School of Engineering, Computing and Mathematics (SECaM), University of Plymouth, Plymouth PL4 8AA, UK; dena.bazazian@plymouth.ac.uk; 3 School of Nursing and Midwifery, University of Plymouth, Plymouth PL4 8AA, UK; marius.varga@plymouth.ac.uk

**Keywords:** augmented reality, indoor navigation, infrastructure-free localisation, multimodal interaction, older adults, semantic graph, spatial anxiety, vision–language models, visual verification

## Abstract

Older adults often need guidance when visiting new buildings for the first time. However, indoor navigation remains challenging due to the lack of Global Positioning System (GPS) availability, visually repetitive corridors, and frequent location failures. This article presents a multimodal indoor navigation assistant that combines graph-based route planning with visual landmark verification to provide step-by-step guidance. The environment is modelled as a directed graph whose nodes are annotated with semantic landmarks, and the graph is constructed primarily from a video of the building, reducing the need for 3D scanners, beacons, or other specialised instruments. Routes are calculated using Dijkstra’s shortest-path algorithm over the semantic graph. During navigation, camera frames are analysed using a restricted vision-language recognition strategy that only considers candidate landmarks from the current and next nodes, reducing false detections and improving interpretability. To increase robustness, a temporary voting mechanism was introduced to confirm node transitions, as well as a hierarchical redirection strategy with local and global recovery. The system is implemented in two modes: handheld mode with visual cues using augmented reality arrows, mini map and voice instructions, and hands-free mode with front camera using voice instructions and keywords. Evaluation involved preliminary technical testing in the United Kingdom followed by formal user validation in Spain. During these trials, participants reported high usability, strong confidence and safety, and increased perceived independence.

## 1. Introduction

Indoor navigation in real buildings is difficult because sensorisation and perception are inherently uncertain, corridors can be visually repetitive, signage is often inconsistent, and decision points amplify the impact of small recognition errors. Although indoor positioning systems and infrastructure-free approaches have been widely studied [1,2,3,4], robust navigation assistance requires more than estimating location when evidence is lacking or ambiguous; it demands explicit state tracking and recovery strategies [5,6]. This challenge is particularly relevant for older adults, for whom disorientation can increase anxiety and reduce perceived independence [7]. In practice, this disorientation appears in everyday activities such as hospital visits, administrative procedures, or participation in social events, which often involve moving through complex buildings with long corridors, multiple intersections, and indistinct visual references [8]. It may also arise when individuals transition to new environments, such as moving into residential care facilities or other unfamiliar settings, where the lack of prior spatial knowledge increases confusion and uncertainty. In these scenarios, loss of orientation can increase stress and reduce perceived autonomy, causing many people to rely on family members or carers to feel safe during the journey [7]. In addition, certain perceptual impairments related to the estimation of spatial properties, for example, depth, width, or personal space, have been associated with functional risks in older populations [9]. Recent meta-analyses corroborate this vulnerability, indicating that older adults exhibit a standardised mean difference of 1.15 in navigation performance compared to younger groups, a deficit that becomes more pronounced in tasks requiring the formation of allocentric cognitive maps [10]. This gap underscores the need for assistive systems capable of externalizing the burden of spatial memory.

Unlike outdoor navigation, where Global Navigation Satellite System (GNSS), commonly accessed through the Global Positioning System (GPS), provides robust global positioning, indoor environments typically lack pervasive and reliable positioning infrastructure. This limitation has motivated extensive research on Indoor Positioning Systems (IPS) and indoor navigation technologies [2]. However, many proposals face adoption barriers in real deployments [11], particularly in public buildings where infrastructure changes, extensive calibration, or specialised equipment may be impractical. To reduce these barriers, infrastructure-free methods take advantage of sensors already embedded in consumer devices through techniques such as visual odometry and (visual-inertial) Simultaneous Localization and Mapping (SLAM) [12]. Nevertheless, indoor navigation is a challenging problem involving localisation under perceptual uncertainty that demands robust solutions to maximize user confidence. Older users may deviate from the suggested route or hesitate at decision points. In many existing systems, such failures are handled implicitly or with limited recovery logic, which can degrade confidence and increase cognitive load when users require reassurance.

This work presents a multimodal indoor navigation assistant designed specifically to support elderly people with sensory impairments or mild cognitive impairment. The proposed system models the environment using a decision-oriented directed graph generated from conventional video recordings, without the need for pre-installed infrastructure such as beacons or 3D scans. Using Dijkstra’s algorithm on this semantic structure, the assistant calculates efficient and comprehensible trajectories, adapted to the demands of built environments. To ensure reliability in complex spaces, the system integrates a context-sensitive visual verification strategy. This approach restricts sensory perception to the expected progress of the route, reducing ambiguity in repetitive indoor environments. It also incorporates a hierarchical recovery mechanism that manages potential inconsistencies through local corrections or global replanning, ensuring the system’s resilience in the face of perceptual uncertainty.

User interaction is managed through a multimodal interface with two modes: a handheld mode, which uses augmented reality and simplified maps, and a hands-free mode, based on voice commands. Both are designed to reduce user anxiety and reinforce their confidence in the assistant, thus increasing their autonomy in unfamiliar environments [7]. Finally, the validity of this proposal has been verified through a two-stage evaluation, comprising technical validation by experts and a study with end users, demonstrating the viability and effectiveness of the system in everyday use scenarios.

The remainder of this article is structured as follows. Section 2 reviews the literature on indoor navigation, assisted orientation and multimodal guidance systems. Section 3 describes the proposed Vertex system, including the semantic graph construction process, route planning and state estimation, visual verification with contextual constraints, augmented reality guidance and the voice assistant. Section 4 presents the experimental setup, including both the pilot study in the UK and the main user-centred validation in Spain. Section 5 presents the results, with particular attention paid to usability and the evaluation of multimodal functions. Section 6 analyses the main conclusions, limitations and implications of the study. Finally, Section 7 concludes the article and outlines future work.

## 2. Related Work

Ageing is often associated with a decline in spatial abilities and a reduction in the acquisition of reliable spatial knowledge, which negatively affects orientation performance in complex environments. In addition, there is high inter-individual variability, and wayfinding can also be influenced by sensory, attentional, and emotional factors, especially in unfamiliar environments [13]. According to experts, perceptual difficulties in interpreting spatial properties and highly homogeneous visual environments can exacerbate wandering and disorientation, linking navigation failures to safety concerns [14]. These findings motivate the development of assistance systems that reduce cognitive load, provide salient cues, and offer reliable confirmation of progress.

The ecological validity of using digital models to predict and assist real-world navigation has been reinforced by Goodroe et al. [15], who showed that performance in a virtual navigation task effectively predicts real-world navigation competence in moderately challenging environments among adults aged 54 to 74.

A wide range of indoor positioning solutions has been proposed, with different trade-offs between accuracy, energy consumption, scalability, interoperability, and deployment cost [1]. Some of the most widespread are based on Wi-Fi signals using fingerprinting techniques, which can offer acceptable accuracy but often require a thorough study of the environment and regular maintenance to remain reliable as signal conditions change [16,17]. In other cases, beacon-based systems are used, for example, Bluetooth, which provide indoor guidance but require the installation of additional hardware and calibration. Recent works attempt to mitigate their maintenance by applying optimisation algorithms, for instance, Particle Swarm, to automate signal thresholding, and calibration [18], yet feasibility remains a challenge in large-scale buildings or those without access to infrastructure [2].

On the other hand, some approaches use detailed geometric reconstructions through Light Detection and Ranging (LiDAR) scanning or other similar techniques. These detailed maps enable advanced egocentric Augmented Reality interfaces that significantly improve user wayfinding performance and reduce user cognitive load compared to traditional 2D maps [19]. However, these solutions often introduce additional acquisition costs and specialised equipment for the 3D scanner, which remains a barrier for scalable deployment in everyday buildings [20]. These limitations reflect a persistent gap between technically feasible indoor localisation methods and solutions that can be deployed and maintained sustainably at scale in everyday buildings.

To reduce dependence on external systems, some infrastructure-free approaches use sensors already present in mobile devices [21]. Inertial navigation systems, particularly those placing sensors on the foot instep, can estimate stride length and orientation with high accuracy by applying Kalman filtering to analyze pitch and roll angles [22]. However, they suffer from inevitable drift over time, often requiring fusion with map constraints or periodic corrections to reset the cumulative error.

To address the drift inherent in low-cost sensors, recent research has explored Deep Learning-based fusion strategies. Ma et al. [23] proposed an LSTM-based algorithm that fuses WiFi fingerprints with Inertial Measurement Unit (IMU) sequences, significantly reducing position error compared to traditional filtering methods. On the hardware side, Ultra-Wideband (UWB) solutions have also matured; Santoro et al. [24] demonstrated a UWB positioning system with “infinite scalability” capable of tracking unlimited assets with high update rates. However, these solutions typically require specific infrastructure (WiFi training phases or UWB anchors) or specialised hardware not universally present in older adults’ smartphones. Consequently, our work focuses on maximizing the utility of standard visual and inertial sensors available in consumer devices.

Other approaches use the camera to estimate displacement using techniques such as visual odometry or SLAM. For instance, recent markerless visual SLAM architectures propose generating abstracted 3D maps composed of keypoints and transmitting them to a remote server [25]. This strategy allows offloading heavy computation to preserve mobile battery and supports multi-user access to the same navigational map, while enhancing privacy by avoiding the transmission of raw video feeds. Such methods allow the device’s movement to be tracked and, in some cases, directions to be displayed using augmented reality. However, these methods often require a prior mapping of the building [26]. Additionally, image-based localisation methods estimate the user’s pose by matching a captured image against a database of reference views. Regarding this, some solutions utilise Deep Learning models, such as Deep Belief Networks (DBN), for scene classification combined with Perspective-n-Point (PnP) algorithms to accurately recover the 6-DoF pose [27]. While effective under controlled conditions, such approaches depend on maintaining an updated visual database and may be sensitive to illumination changes, occlusions, and repetitive indoor corridors.

To increase reliability, some systems incorporate artificial markers such as QR codes or specific signs placed manually in the environment [28,29]. Although this solution can be effective, especially for resetting the cumulative error generated by odometry sensors in low-density setups, it requires physically modifying the building and depends on permits, maintenance and long-term acceptance, which is not always feasible in some types of facilities or public buildings.

Recent work has also explored semantic representations for indoor navigation, including connectivity graphs enriched with building semantics. For instance, semantics-based approaches using Industry Foundation Classes (IFC)-derived graphs demonstrate flexible object-to-object and space-to-space rather than coordinate-based point-to-point navigation, avoiding computing-intensive geometry processing, but assume access to detailed building information models that are not always available [30]. In addition, computer vision has also explored representing videos as graphs. Instead of analyzing the video frame by frame, some models create a graph where the nodes are detected objects and the connections represent relationships between them, allowing for better reasoning about what happens over time. One example is Video Graph Transformer (VGT), which uses dynamic graphs to understand relationships and movements between objects in video question-answering tasks [31]. Inspired by these ideas, our approach uses a video tour of the building to construct a compact graph with important locations and semantic landmarks, without the need to reconstruct a complete 3D model.

In the domain of semantic mapping, recent advances have moved beyond static object detection toward Open-Vocabulary 3D Scene Graphs. Works such as ConceptGraphs [32] and Hierarchical Open-Vocabulary Scene Graphs (HOV-SG) [33] demonstrate the potential of fusing vision-language features directly into 3D structures. These methods allow for querying environments using natural language without predefined class labels. Parallel to these visual approaches, the construction industry has explored deriving navigation graphs directly from Building Information Models (BIM). For instance, Zhu et al. [30] introduced NAVI-graph, a semantics-based network extracted from IFC files that prioritises space-to-space connectivity over metric geometry. While these approaches provide rich data, they often rely on pre-existing digital twins or intensive 3D reconstruction pipelines. In contrast, our approach prioritises rapid deployment in unmapped buildings by extracting a topological skeleton directly from a monocular video walkthrough.

This trend toward semantics has recently expanded into Open-Vocabulary Functional Scene Graphs, which model not only object presence but also interaction affordances [34]. Vertex adopts a similar philosophy by selecting “landmarks” based on their functional utility for navigation decisions rather than mere visual salience.

Among the different modalities commonly used as guides for indoor navigation, it is common to combine visual, auditory, or multimodal feedback. Augmented reality (AR) overlays, such as arrows or virtual paths, can improve orientation by placing directions directly over the user’s view, reducing the need for attention switching between device and environment [20]. On the other hand, audio guidance can facilitate hands-free use, but language-based instructions can impose a greater cognitive load in complex situations, often resulting in slower navigation times compared to visual cues. Furthermore, the effectiveness and preference for each modality may vary depending on the user profile and context (familiarity with the environment, spatial complexity, level of load, etc.), which justifies comparing different modes of interaction [4,35]. In this regard, it is important to note that cognitive load and spatial anxiety interact, anxiety can increase under conditions of high load and can also partially mediate the relationship between load and navigation performance in both egocentric and allocentric tasks [7,36]. Research suggests that combining egocentric cues, like guideposts, with allocentric aids, like 3D layout models, is particularly effective for developing cognitive maps and reducing this mental workload. In order to design interfaces that provide clear and confirmatory feedback, reducing uncertainty, our work provides an assistant that integrates voice instructions, directional signals in AR, and a compact view of the map. We also evaluate a hands-free mode focused primarily on interaction and guided by voice, and a mode with visual support in addition to voice guidance, to analyze how these alternatives affect usability and perceived safety.

To make the distinction with prior indoor navigation systems explicit, Table 1 summarises the main differences between Vertex and representative approach families. While existing solutions often depend on dedicated environment instrumentation, computationally heavy dense 3D reconstructions, large image databases, or pre-existing digital building models, Vertex is designed as a rapidly deployable, infrastructure-light assistant. It leverages standard mobile sensors to construct a lightweight semantic graph directly from a monocular video walkthrough. Technically, our architecture performs context-constrained landmark verification using Vision-Language Models (VLMs) and incorporates temporal voting and hierarchical recovery mechanisms to ensure robustness under perceptual uncertainty. Ultimately, rather than solely maximising metric localisation accuracy, the main contribution of Vertex is its user-centred design tailored for older adults: it combines semantic interpretability with multimodal feedback (voice instructions, AR cues, and map views) to enhance perceived safety, confidence, and independence in unfamiliar buildings.

In summary, the proposed work differs from the reviewed literature in terms of its practical implementation model, its technical design and its user-centred approach. Vertex enables fast deployment using only sensors commonly found in standard mobile devices, thereby avoiding the need for additional infrastructure or pre-existing building models. Rather than relying on dense geometric reconstruction, full metric mapping or large reference image databases, the system constructs a lightweight semantic graph. The proposed architecture is designed for decision-making based on a video trajectory and performs context-constrained landmark verification using vision-language models. Furthermore, it incorporates temporal voting and hierarchical retrieval mechanisms to improve robustness in the face of perceptual uncertainty. Finally, unlike other more general systems, the proposed approach is specifically oriented towards older adults, combining voice, AR cues and map-based feedback to improve perceived safety, confidence and independence during indoor navigation.

## 3. Materials and Methods

To address the challenges of infrastructure-free indoor navigation for older adults, a multimodal navigation framework that combines topological reasoning with semantic visual verification has been developed. This system relies on a decision-oriented semantic graph constructed from a simple video walkthrough which models the environment not as a metric space, but as a sequence of navigational decisions and identifiable landmarks.

**Figure 1:**
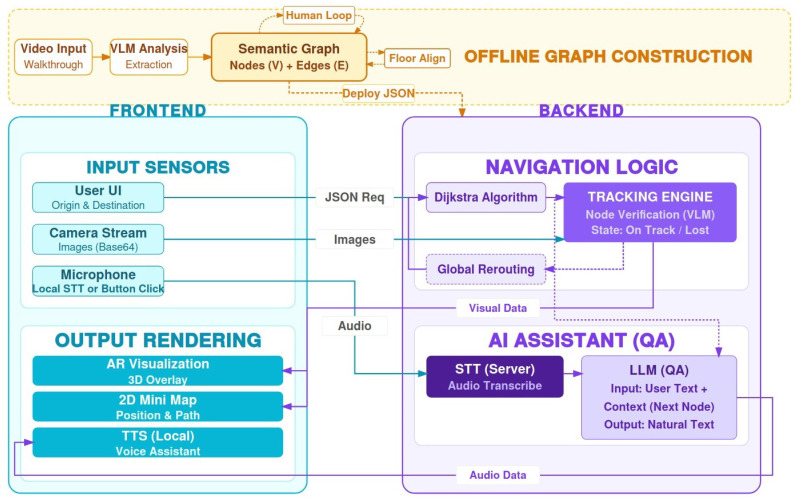
System architecture.

The navigation assistant presented works using a client–server model designed to respond quickly and smoothly. On the one hand, the client, developed in Unity (Unity Technologies, San Francisco, CA, USA, https://unity.com) for mobile devices, serves as the user’s “sensory interface”, capturing images with the camera, using the phone’s sensors to calculate orientation, and managing communication with the user through voice instructions and visual signals. On the other hand, the Server, programmed in Python (Python Software Foundation, Wilmington, DE, USA), acts as the reasoning engine, responsible for deciding the best route, processing visual information, and managing the user’s progress along the way. The complete interaction between these components is illustrated in Figure 1.

One of the main innovations of this system is the use of Vision Language Models (VLMs) to identify where the user is without the need for prior training (zero-shot localisation). The ability of multimodal Large Language Models to support spatial orientation has been validated by Halama et al. [37], who showed that models such as GPT-4 Vision (OpenAI, San Francisco, CA, USA) can interpret complex scenes for navigation. Our system adapts this capability through context-restricted verification to minimise hallucinations. Instead of teaching the AI photos of every corner of a building, the server uses a context-guided verification strategy, only analysing images to search for specific objects that the system’s internal graph indicates should be nearby. To avoid errors or confusion, a state machine (a control logic that manages transitions between tracking, validation, and recovery phases) has been implemented using a temporal voting system; this means the system needs to confirm the same evidence in multiple consecutive frames before validating a change of position, automatically activating search plans if the user gets lost. The main elements of the system are detailed in the following subsections.

### 3.1. Decision-Oriented Semantic Graph

To overcome the barriers to adoption of visual SLAM-based systems, which typically require dense point clouds and environment-specific retraining, we propose a lightweight and scalable mapping methodology. With this objective, we model the interior environment of the building as a semantic topological graph G=(V,E), where absolute metric accuracy is subordinated to semantic interpretability and clarity in decision-making. Formally, we define the graph using a set of nodes *V* representing decision points (such as intersections or final destinations) and a set of directed edges E⊆V×V modelling navigable transitions. We model each node $v_{i}$ as a semantic tuple:

(1) $$v_{i}=\langle T_{i},p_{i},f_{i},L_{i}\rangle$$

where $T_{i}$ contains textual labels for speech synthesis, $p_{i}$ represents relative spatial coordinates, $f_{i}$ denotes the floor index or level identifier, and $L_{i}=\left\{l_{i,1},\ldots ,l_{i,k}\right\}$ is a set of natural language landmark descriptors. These landmarks consist of descriptions of distinctive objects at that location, allowing the system to perform visual checks without relying on pre-calculated feature descriptors or models trained specifically for the building.

On the other hand, each directed edge $e_{ij}\in E$ connecting two nodes $v_{i},v_{j}$ is defined as a tuple:

(2) $$e_{ij}=\langle w_{ij},I_{ij}\rangle$$

where $w_{ij}\in R^{+}$ represents the physical distance or metric weight between nodes, and $I_{ij}$ contains the specific natural language navigation instruction provided to the user during traversal. This instruction can be dynamically reformulated during navigation using a Large Language Model (LLM); however, storing a predefined instruction for each transition reduces latency the first time the system queries that edge.

The graph construction process avoids costly object detector training, employing instead a supervised zero-shot semantic generation strategy. The workflow begins with the recording of a continuous tour of the building, which is processed by a Visual Language Model using a few-shot learning technique. In the evaluated deployments, this offline acquisition stage was performed using a continuous walkthrough video of approximately 4.5 to 6.5 min for two-floor buildings. During recording, the operator traversed the building in all relevant directions, covering the main circulation paths and ensuring that each salient landmark was observed at least once. The purpose of this video is not to build or retain a dense frame-level map, but to provide sufficient semantic coverage for VLM-based segmentation into decision nodes, transitions, and landmark descriptors. After this stage, the runtime representation stored by the system is a compact semantic graph containing node labels, edge connectivity, normalized coordinates, landmark descriptions, and predefined navigation instructions. This is consistent with the paper’s framing of the approach as a lightweight semantic graph. Using an extensive prompt that includes examples of valid JSON structures, we instruct the model to segment the video into logical nodes and automatically extract both the connection topology and the visual landmarks most appropriate for human navigation. Since the system is designed for vulnerable users, we subsequently integrate a “Human-in-the-Loop” validation stage. In this phase, the structure proposed by the VLM is reviewed to ensure the safety of the routes and refine the visual descriptions if they are ambiguous, thus combining the efficiency of generative AI with the reliability of human supervision.

Finally, to provide the system with spatial awareness without sacrificing its topological flexibility, the nodes of the graph are aligned by validating them against the image of the building’s floor plan. Using a lightweight annotation script, each node is injected with its normalised coordinates $p_{i}=\left(u_{i},v_{i}\right)\in {\left[0,1\right]}^{2}$ on the building map. This spatial data is essential for computing the path cost required by the Dijkstra-based planning algorithm and for dynamically orienting the Augmented Reality arrow relative to the device’s magnetic north during navigation. This hybrid methodology enables rapid deployment in new environments while maintaining the necessary infrastructure for reliable multimodal indoor assistance.

### 3.2. Route Planning and State Estimation

Once the semantic graph has been defined, navigation is divided into two phases: first, the route is calculated, and then the step-by-step guidance begins, which is supervised by the system through a deterministic state machine designed to prioritise temporal consistency.

Given a source-destination tuple $\left(v_{start},v_{goal}\right)$ selected by the user via the visual interface, the system computes the best path $P=(v_{1},\ldots ,v_{k})$ using Dijkstra’s shortest path algorithm. The algorithm iteratively expands the node with the lowest accumulated traversal cost, ensuring that the resulting path is optimal with respect to the defined edge weights. The cost function is defined solely in terms of g(n), which represents the cumulative cost from the origin to the current node, computed as the sum of edge weights w(e) that approximate the physical distance or estimated transit time between nodes. This method corresponds to a uniform-cost search strategy, prioritising robustness and guaranteeing optimality under the available graph cost representation.

Although the search formulation is compatible with A*-based planning, no heuristic term h(n) was incorporated in the evaluated deployment. Given the relatively small size of the navigation graphs in the tested buildings (two floors, limited decision nodes), the computational cost of uniform-cost search is negligible. Avoiding an explicit heuristic simplifies validation and guarantees optimality without requiring additional assumptions about spatial admissibility in indoor environments, where Euclidean distance may not perfectly reflect traversal cost, for instance in the case of stairs or constrained corridors. Nevertheless, the availability of normalized node coordinates enables straightforward integration of an admissible spatial heuristic in future large-scale deployments.

At each step of the route ($v_{i}\to v_{i+1}$), the system uses predefined instructions stored directly in the graph. This design aims to minimise latency: by avoiding generating text in real time for each step, immediate delivery of the instruction and a smooth navigation experience are guaranteed. However, to provide flexibility, an auxiliary AI (LLM) is incorporated. If the user has questions such as “should I go straight?”, the model generates an enriched explanation using the context of the graph and visual references from the environment.

During the guidance phase, the system operates in an active perception loop designed to tolerate uncertainties. User localisation follows a priority hierarchy evaluated at each inference cycle, employing a cumulative voting scheme that requires repeated visual evidence before confirming a transition. Given that sequential image capture for landmark validation may introduce noise, for example motion blur or partial occlusions, the system avoids instantaneous state changes to maintain temporal consistency. The state update logic $S_{t}$ operates as follows:

- Tracking: The system first evaluates whether the user has reached the expected next node ($v_{next}$). A positive visual detection (r>τ) must be maintained for a minimum number of consecutive cycles before updating the route index (i←i+1) and issuing the next instruction. This filters out momentary false positives caused by transient visual artefacts or ambiguous observations.
- Rerouting: If the landmarks of the current arc are not detected, the vision model analyses an expanded set of candidate nodes $C_{search}$, defined by the immediate neighbours within a radius δ of both the current and the next node:
  (3) $$C_{search}=\left(N_{\delta }\left(v_{curr}\right)\cup N_{\delta }\left(v_{next}\right)\right)\setminus \left\{v_{curr},v_{next}\right\}$$
  Note that the current and next nodes are explicitly excluded from this set, as they are already evaluated in the primary tracking stage. This separates the confirmation of the route from the detection of deviations. For our prototype, we configured a search radius of δ=1 hop, limiting the search space to immediate adjacency to maintain real-time performance. If an off-route node $v_{off}$ is identified, a “deviation vote” counter is incremented. Only if the evidence persists for a sufficient number of cycles is the user declared to have deviated, and the optimal route from $v_{off}$ to the destination is recalculated using Dijkstra’s algorithm. This strategy allows the system to identify whether the user has deviated to an adjacent corridor or has advanced faster than expected.
- Global localisation: In situations where no matches occur with the local neighbourhood for an extended period, the system assumes the user is disoriented. In this state, the search is expanded to the entire graph. If a positive localisation with high confidence is achieved in consecutive instances, the system restarts navigation from the newly inferred location. Additionally, global localisation can be manually triggered by the user via a button in the interface.

If localisation still cannot be established after the global search stage, the system does not continue issuing potentially misleading guidance. Instead, the interface prompts the user to retry the environment scan from a nearby distinctive decision point, such as an intersection or staircase, where semantic landmarks are more likely to support successful re-localisation. In this formulation, localisation is estimated as a discrete topological state over the semantic graph rather than as a continuous 2D/3D pose. Accordingly, navigation error is operationalised as an incorrect node assignment, a false or delayed node transition, or an off-route state, inferred from the landmark-matching ratio and the temporal voting mechanism. Because the evaluated prototype does not estimate continuous metric position and the present study did not include external ground-truth tracking, coordinate-based accuracy measures such as RMSE were not reported here.

### 3.3. Context-Constrained Visual Verification

In order to mitigate latency and hallucinations that general-purpose Vision-Language Models may exhibit, the system does not just request a generic scene description. Instead, it implements a context-constrained verification strategy. The backend injects into the model’s prompt a closed list of candidate objects $O_{cand}$, expected at the current location, and instructs the model to act as a discriminative filter. The visual verification process is executed in three stages: context injection, text normalisation, and fuzzy matching.

First, the system sends the captured image $I_{t}$ to the VLM along with the textual labels of objects associated with the candidate nodes ($C_{search}$). The prompt design implements a closed-set validation mechanism. The model is instructed to restrict its output space exclusively to the provided tokens, explicitly penalising the generation of external objects (negative constraint) and standardising the absence response by returning the token “none” when none of the listed objects are detected.

Both the graph labels and the model’s response then undergo preprocessing that includes accent removal, conversion to lowercase, stopword filtering, and basic lemmatisation, for example plural reduction, in order to maximise semantic matching.

Given that the model’s output may present morphological variations with respect to the stored labels, a similarity coefficient based on token intersection is computed. For a candidate node *v* with reference tokens $T_{ref}$ and detected tokens $T_{det}$, the confidence ratio *r* is defined as

(4) $$r\left(v,T_{det}\right)=\frac{|T_{ref}\cap T_{det}|}{|T_{ref}|}$$

A node is considered visually recognised only if this ratio exceeds an empirical threshold τ, which allows validating compound objects, such as “wooden door”, even when detection is partial. For this implementation, we set τ=0.5 to validate detections while allowing for minor descriptive variations.

### 3.4. Sensor Fusion and Augmented Reality Guidance

**Figure 2:**
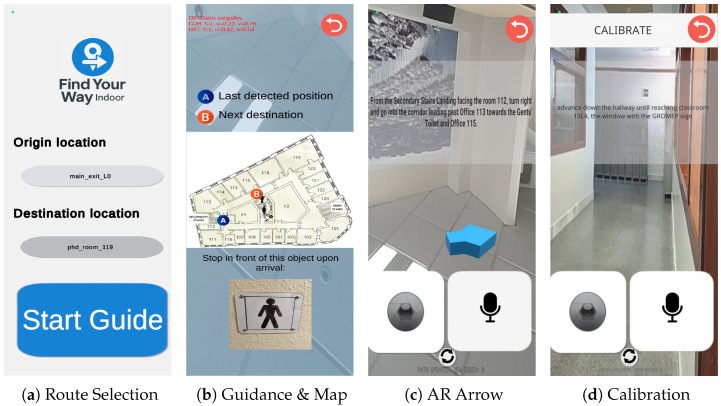
User Interface Design overview showing the navigation workflow.

To provide intuitive directional guidance in indoor environments, the system incorporates an Augmented Reality overlay interface, as shown in Figure 2. A 3D directional arrow points the user towards the next topological node ($v_{next}$). The orientation of this indicator is controlled by a heading estimation pipeline that prioritises the device’s magnetometer to obtain an absolute heading, and falls back to the gyroscope when the OS-reported heading accuracy is unavailable or ≥5°. Therefore this design avoids dependencies on ARCore/ARKit to maximise coverage on legacy and low-end smartphones, which are more prevalent in the target demographic.

Given the noisy nature of consumer-grade IMU sensors indoors, raw data is processed through a circular sliding-window smoothing algorithm. Standard linear averaging fails at the compass wrap-around point (where $359^{\circ }$ meets $0^{\circ }$). To resolve this, our system maintains a First-In, First-Out (FIFO) buffer of the last N=10 yaw readings. The smoothed orientation ${\bar{\psi }}_{t}$ is calculated by averaging the minimum angular differences relative to the first sample in the current window $\psi_{base}$:

(5) $${\bar{\psi }}_{t}=\psi_{base}+\frac{1}{N}\sum_{i=1}^{N}\min\_\mathrm{delta}\left(\psi_{base},\psi_{i}\right)$$

This approach mitigates high-frequency jitter while preserving rotational continuity during direction changes. Furthermore, to mitigate magnetic interference typical of reinforced concrete buildings, the system includes a calibration mechanism. At the beginning of the route, the user can align the camera with a known reference point and trigger a recalibration procedure. This process computes a yaw correction offset ($\Delta_{yaw}$), which is subsequently applied to all incoming frames.

Complementing the AR view, a context-aware 2D map provides global spatial awareness. Unlike static images, this module dynamically renders the topological state by mapping normalised graph coordinates (u,v)∈[0,1] to screen space, displaying the Current Node and Next Target on the corresponding floor plan. For destination verification, the interface can optionally retrieve and display a reference image of the target point of interest associated with the destination node, as long as it was incorporated into the system during the offline building adaptation phase and stored locally. Following a cognitive load reduction strategy, this map appears automatically during node transitions to aid orientation and is automatically hidden after a timeout during straight segments.

### 3.5. Voice-Driven Contextual Assistant

In order to support hands-free interaction, the system integrates a bidirectional voice interface. The pipeline is activated through a wake-word detection module, which relies on a locally deployed keyword recogniser running on the mobile device. Once the keyword “Vertex” is detected, the system automatically triggers the capture of the voice query, which is then transmitted to the backend, where it is transcribed using an on-device instance of OpenAI’s Whisper-small speech-to-text (STT) model [38] via Faster-Whisper (OpenAI, San Francisco, CA, USA) [39]. This approach ensures privacy and low latency by avoiding external dependencies at this stage. Voice-based queries can also be initiated through a dedicated button in the user interface.

Unlike standard voice assistants, this module is context-aware. When the user poses a question (e.g., “Where is the elevator?”), the backend injects the current navigation state $S_{t}=\left\{v_{curr},v_{next},d_{goal}\right\}$ into the LLM’s system prompt. This allows the model to generate relative spatial answers, for example “It is straight ahead, beyond the glass door”, rather than generic definitions. The response is finally synthesised into speech using the native Android Text-To-Speech (TTS) engine, providing an accessible feedback loop for visually impaired users.

### 3.6. System Architecture and Implementation Details

The system implements a decoupled client–server architecture, designed to offload computationally intensive tasks, such as VLM inference and route planning, from the mobile device to a dedicated backend. The development environment is therefore divided into two main blocks.

The reasoning engine that acts as backend is built on Python 3.10 using the FastAPI framework. It is hosted on a workstation equipped with an NVIDIA GeForce RTX 3060 GPU (Nvidia, Santa Clara, CA, USA) to accelerate local inference of the Whisper model. To minimise access times, the semantic graph structure is managed in memory.

The mobile application, developed in Unity 2022.3 (LTS), was used on a Google Pixel 3a during the evaluation as the client device, and functions as a sensor hub. It leverages Unity’s native sensor APIs for sensor fusion (magnetometer/gyroscope) and WebCamTexture for image capture, aggregating these heterogeneous data streams into a unified JSON payload for each inference cycle.

Communication is established through a RESTful API. Given that navigation is an inherently stateful process requiring the persistence of a historical trajectory, but the HTTP protocol is stateless, and the system implements an In-Memory Session Manager. Navigation state is stored in a volatile structure to increase speed. This design prioritises low latency over long-term durability, a decision justified by the ephemeral nature of the data: once the destination is reached, state metrics (route indices, voting counters) become obsolete.

The data flow is optimised for real-time interaction. Visual data is encoded as Base64 strings and encapsulated within the JSON object, while audio buffers are attached to the same request. This ensures that the backend receives a synchronised snapshot of the user’s context (visual field + verbal intent). After processing, the API response transports not only the textual instruction but also semantic metadata, such as the updated route index and normalised coordinates (u,v) for dynamic map rendering.

A hybrid computation strategy is implemented for the audio subsystem. While complex speech recognition (STT) is performed on the server using a local Whisper model (ensuring privacy and speed), text-to-speech synthesis (TTS) is delegated to Android’s native engine (android.speech.tts) via AndroidJavaObject. Although less natural than server-side generation, this approach eliminates network latency for auditory feedback.

To ensure reproducibility, Table 2 details the specific configurations of the AI models.

It is worth noting that the VLM model temperature was strictly set to 0.0 with a limit of 60 tokens to minimise hallucinations and enforce concise outputs. For the audio subsystem, the selection of faster-whisper-small executed on GPU enabled inference times below 200 ms for typical navigation commands, balancing accuracy with real-time interactivity.

## 4. Experimental Setup

To validate the usability and perceived impact of the proposed indoor guidance assistant, an internal feasibility test was carried out in the United Kingdom and a formal user validation study in Spain under ethical approval from the UPV Research Ethics Committee (reference P11_26-09-2025). This design was selected to progressively refine the interaction flow based on feedback obtained in the first informal prototype test in the UK. The UK pilot was not intended to assume environmental equivalence with the Spain site. Rather, it served to refine general interaction and robustness parameters of the system. The Spain study was conducted with a site-specific semantic graph and local building adaptation generated from the Spain building itself.

Moreover, it should be noted that the two evaluation phases were conducted in different languages, the UK pilot used English voice instructions and interface text, while the Spanish validation was conducted in Spanish, with landmark descriptions and TTS configured accordingly. The system supports this kind of language adaptation at the graph and prompt levels without structural changes. Therefore, differences in voice-related scores between the two phases likely reflect both the technical refinements applied after the pilot and the benefit of receiving instructions in the participants’ native language.

In addition, the evaluation was conducted under naturally varying real-use conditions, including sessions performed at different times of day and in different buildings with distinct layouts, orientations, and window configurations. Consequently, the system was exposed to both natural daylight and artificial indoor lighting during the pilot and validation phases.

All participants provided informed consent prior to participation and were free to withdraw at any time. Data were anonymised and analysed in de-identified form, and only aggregated results are reported. Moreover, camera frames were processed strictly for the purpose of verifying predefined indoor landmarks during navigation and were not stored after processing. Raw data were processed exclusively for research purposes, stored securely with restricted access, and were not shared between the United Kingdom and Spain.

It is worth noting that the evaluation has been designed as a System Validation Study. By focusing on technical feasibility and acceptability in a vulnerable population, the design prioritises within-group evaluation and comparison with industry usability standards, ensuring participant safety against unassisted navigation tasks that could generate stress or disorientation.

Under this framework, the evaluation focuses on the following hypotheses:

**Hypothesis** **1 (Usability and Robustness).** *Older adults will report usability above the acceptability threshold (SUS>68).*

**Hypothesis** **2 (Voice as Key Factor).** *Perceived clarity of voice instructions will correlate strongly with overall SUS score, supporting the design centred on the auditory channel as the primary guidance method.*

**Hypothesis** **3 (Impact and Multimodal Perception).** *The multimodal interface will be valued positively, significantly increasing the user’s sense of security and perceived independence in complex environments.*

### 4.1. Pilot Study (UK)

The first evaluation phase consisted of a preliminary technical pilot study carried out in the Reynolds building at the University of Plymouth (United Kingdom). This phase had a formative character and focused on debugging the system, identifying critical usability points and perform a formative evaluation of the interaction flow before engaging with vulnerable users. The Reynolds building was strategically selected for presenting repetitive and symmetric interior structures across two floors, while being sufficiently small for a first proof of concept; these characteristics enabled initial testing of the system’s temporal voting and hierarchical rerouting mechanisms.

In total, n=13 volunteer participants were recruited among university staff, with a mean age of 59.36 years and standard deviation (SD) of 12.71, who acted as coworkers. Although this convenience sample presented a wider age range than the target population, it was deemed sufficient for a formative assessment of system stability and interaction flow prior to deployment with vulnerable users. In this internal feasibility test, participants navigated to various destinations using two interaction modes: a hands-free device hanging from the neck with predominantly auditory guidance, and a handheld device, combining the AR arrow, mini-map, and voice instructions.

After completing the tasks, participants evaluated the system through a questionnaire that integrated the System Usability Scale (SUS) as a usability indicator, Likert-type questions about key components (voice clarity, AR arrow, route selection, and voice interaction), and an open comments section. The results obtained in this phase enabled crucial technical and interface adjustments before proceeding to formal validation with the target population in Spain.

### 4.2. Main Study: User-Centered Validation (Spain)

The main validation was conducted in a public building in Spain with n=21 older adults with a mean age of 67.33 years (SD = 3.24). This phase constituted the core of the end-user evaluation, enabling the assessment of not only the system’s technical robustness, but also its real impact on perceived security and user independence within the participants’ cultural and social context.

It is important to note that the British and Spanish samples differ both in their age distribution and in their experimental purpose. The UK sample (mean age = 59.36; SD = 12.71) was drawn from a pilot study involving university staff, designed to fine-tune the system and refine the interaction workflow prior to validation with the target population. This initial phase involved both older lecturers, who were closer to the end-user profile, and middle-aged researchers, which explains the greater dispersion observed. In contrast, the Spanish sample (mean age = 67.33; SD = 3.24) consisted of older adults recruited through the UPV Senior University programme, making it a more accurate representation of the study’s target population. In this case, the majority of participants were concentrated within a more homogeneous age range, approximately between 65 and 75 years. These differences should therefore be understood as a consequence of the study’s sequential design rather than as a direct comparison between equivalent cohorts.

**Figure 3:**
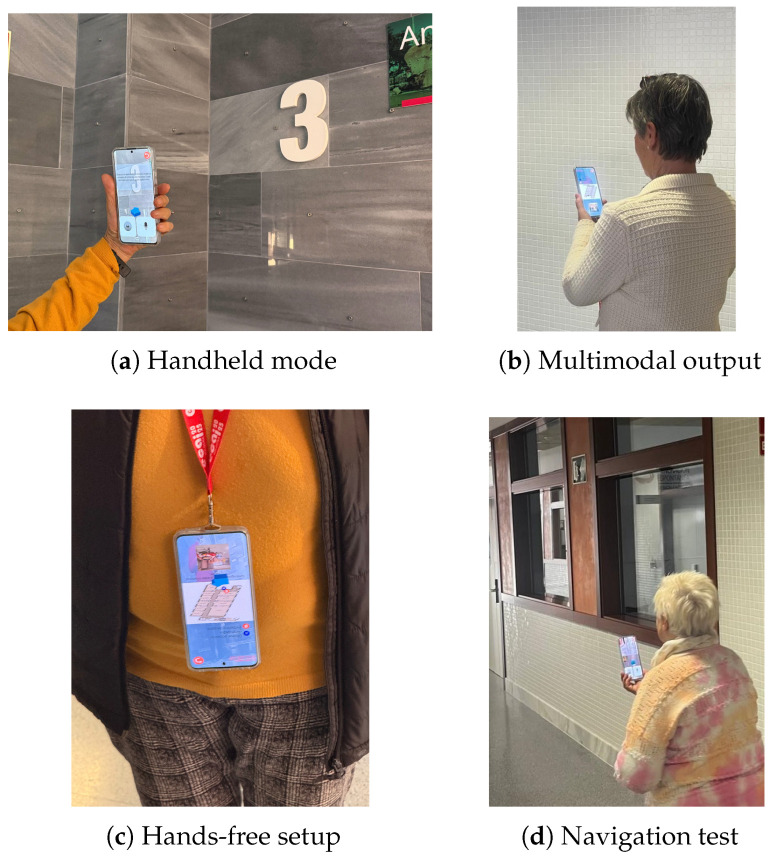
User validation setup. (**a**) Visual landmark verification. (**b**) Interaction switching (voice/map). (**c**) Lanyard setup. (**d**) Participant in the test circuit.

Participants in the Spanish study attended the Georgina Blanes building at the UPV campus in Alcoy, where the test was conducted on floors 2 and 3 of the building, following a procedure similar to that applied in the preliminary tests in the United Kingdom. Users were initially summoned to a room, from where they were requested to navigate to the exit and to different points in the building, using the navigation system with voice instructions, visual aids, map, and augmented reality indications. Similarly, both usage modalities were evaluated (device handheld and hanging around the neck). Finally, the same SUS questionnaire was administered, along with the same additional specific questions about concrete functionalities of the application. A visual representation of the experimental validation process can be seen in Figure 3.

## 5. Results

A total of 34 participants evaluated the system across two distinct phases: n=13 university staff members and colleagues participated in a preliminary technical pilot in the United Kingdom, aimed at assessing system stability and obtaining formative feedback, and n=21 older adults participated in the formal validation study in Spain.

The technical pilot conducted in the United Kingdom was instrumental in adjusting the architecture components described in Section 3. Specifically, the feedback obtained enabled calibration of the circular buffer-based low-pass filter to stabilise the orientation signal and reduce vibration of the Augmented Reality arrow. Likewise, the active zones of the touch interface were redefined to prevent accidental application closures, and the readability of graphic elements was optimised. Finally, the voting scheme parameters and confidence thresholds for semantic object verification were refined, thus ensuring the robustness and safety necessary for deployment in the final validation in Spain.

Usability was evaluated using the System Usability Scale (SUS), a standardised 10-item questionnaire with responses on a scale of 1 to 5 that transforms into a single score between 0 and 100 (0 = worst perceived usability, 100 = best). As a widely used interpretative reference, a score around ≈68 is usually considered the “average” value in SUS studies; above this value, the system is perceived as more usable than average.

In the formal validation in Spain, participants reported a mean SUS of 87.50 (SD=17.57). In the technical pilot in the United Kingdom, the mean was 78.39 (SD=17.34) (Table 3). The SD values reflect that, although the overall rating was high, there was variability amongst participants, something to be expected in tests conducted in real building environments.

To facilitate interpretation, in addition to the mean, the percentage of participants who exceeded usual thresholds is reported. In the technical pilot (United Kingdom), 64.3% of responses with computable SUS obtained a score ≥68, and 50% reached ≥80. In the validation study (Spain), 75% reached ≥80, which shows that, following the changes applied based on feedback from the UK tests, user perception, which was already good in the UK, increased further in the tests with real users in Spain. Furthermore, this pattern indicates that the majority perceived the system as usable and that a relevant proportion rated it in a high range, typically described as good–excellent.

An exploratory analysis using Welch’s *t*-test revealed no significant differences between the SUS scores from the initial feedback tests in the UK and the actual usability studies in Spain (p=0.21). Prior to applying Welch’s *t*-test, normality of SUS score distributions was assessed using the Shapiro–Wilk test. Results indicated no significant departure from normality in either the UK pilot (W = 0.920, *p* = 0.216) or the Spanish validation sample (W = 0.857, *p* = 0.111). To further ensure robustness given the small sample sizes, a non-parametric Mann–Whitney U test was conducted as a complementary analysis (U = 50.0, *p* = 0.706), converging with the parametric result and yielding the same substantive conclusion as the parametric analysis, the absence of significant differences between groups. This absence of statistical divergence is especially relevant given the differences between the cohorts (technical staff versus target population, although both had a high mean age). This suggests a high degree of system generalisation, indicating that the interface is sufficiently intuitive to maintain its perceived performance across different user profiles.

The SUS can be decomposed into two components, usability (8 items) and learnability (2 items), where the latter is reported on a scale of 0 to 20. In the tests with real users, the learnability score was high, 15.18/20, which suggests that participants perceived the system as relatively easy to learn even on first use. For descriptive statistics, 95% confidence intervals around the mean were calculated using the t distribution.

We shall now focus on the tests with users in Spain as they represent the formal validation of the proposal with the target population (older adults), carried out under a strict ethical protocol and using the refined and stable version of the system following the technical improvements from the pilot. In these tests, the internal consistency of the scale was solid, obtaining a Cronbach’s alpha of α=0.91 calculated after recoding the reversed SUS items to align the direction of the scale (for negative items, $x^{\prime }=6-x$).

### Evaluation of Multimodal Functions

The 5-point Likert scale items enabled a detailed analysis of specific system components. Table 4 shows the questionnaire results for each modality, which indicate that the main navigation logic was effective, with particular emphasis on the ease of initiating a route and the clarity of voice instructions.

Voice instructions received consistently high ratings (4.58/5 in the final validation; 4.00/5 in the technical pilot). In this context, Spearman’s correlation was used to examine whether higher scores on specific system features were associated with higher overall usability (SUS) scores. A strong association was found between voice clarity and SUS (ρ=0.74, p=0.037), and ease of route selection showed the highest relationship (ρ=0.77, p=0.024), pointing to the overall experience depending mainly on initiating the route without friction and receiving clear voice instructions.

The improvement observed across most items between the pilot and the validation phase appears to result from a combination of iterative technical refinements and contextual differences between the two evaluation settings. Route selection, for instance, remained consistently highly rated in both phases (4.33 in the UK and 4.42 in Spain), suggesting that the origin–destination interface was already intuitive in the pilot version and therefore required only minor adjustments. Although some small refinements were introduced after the UK phase, such as improving label clarity and dropdown behaviour, these changes do not seem to have substantially altered users’ overall perception of this function. Voice clarity showed the largest absolute increase, rising from 4.00 to 4.58. This improvement likely reflects the combined influence of several factors. The system itself had been refined after the pilot phase, including adjustments to the phrasing and delivery of spoken instructions in response to qualitative feedback indicating that some messages were too ambiguous at decision points. These changes likely contributed to a clearer and more reassuring perception of voice guidance in the final study. Map usefulness also improved notably, from 3.53 in the pilot to 4.25 in the validation phase. In the UK pilot, some participants reported difficulties in interpreting the 2D minimap while walking. Following this feedback, the map module was adapted so that it appeared primarily during node transitions and remained hidden during straightforward segments, while still allowing users to open it manually by tapping the arrow, thereby reducing visual clutter and making the map more context-sensitive. A particularly marked change was observed in the usefulness of the AR arrow, which increased from 3.40 to 4.25. This improvement is consistent with the qualitative findings from the UK phase, where user opinions were strongly divided due to perceptible jitter and instability in the directional cue. After the pilot, the smoothing algorithm based on the circular buffer was recalibrated, reducing this instability. The subsequent improvement in ratings suggests that AR directional assistance can be highly effective for older adults when the guidance signal is sufficiently stable. By contrast, voice interaction showed a slight decrease, from 4.00 to 3.92, although this variation is small and does not appear substantively meaningful Even so, the overall score remained clearly positive, indicating that voice interaction continued to be well accepted as part of the multimodal guidance experience.

**Figure 4:**
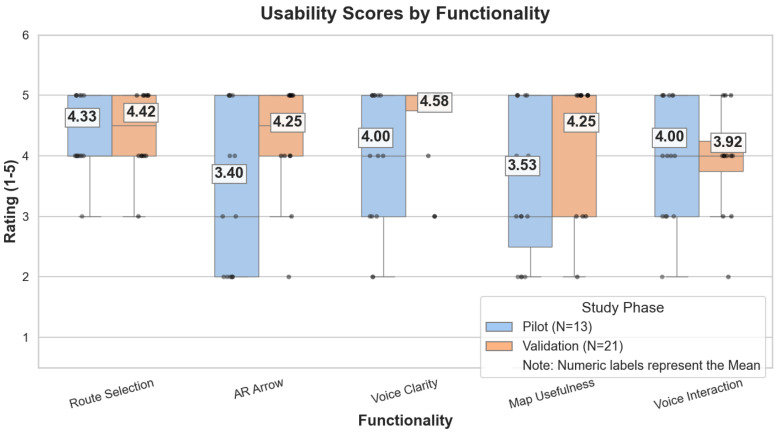
Comparative analysis of user ratings between the formative Pilot phase and the final Validation phase.

Additionally, Figure 4 shows the distribution of user ratings for the five system functionalities evaluated in the questionnaires. These results evidence the positive impact of the technical refinements implemented following the pilot phase, demonstrating a clear improvement in user perception across most categories during the final validation.

In the tests with the target group, the impact items, aligned with the system’s objective, showed particularly high values. Participants reported a high sense of perceived safety when following the instructions, with a mean score of 4.67/5 (SD = 0.50, 95% confidence interval (CI) [4.28, 5.05]), and a high perception of support for independence, that is, the perceived ability to visit new buildings without a companion, with a mean of 4.89/5 (SD=0.33; 95% CI [4.63, 5.15]).

Regarding the mode of use, the predominant tendency was to carry the device in hand (66.7% in Spain, with many users attributing this to habit, especially those who frequently used smartphones), although a significant proportion of participants preferred hands-free alternatives or mixed use. In the United Kingdom, in the preliminary tests, the percentage in favour of carrying it in hand was 53.3%; this lower percentage may be due to the difference in buildings (the UK one being less crowded) and the improvement in the interface and AR visual guidance between the UK tests and those in Spain.

## 6. Discussion

The results presented in this study validate the technical feasibility and acceptance of the multimodal navigation system in an older adult population, demonstrating cross-cultural consistency between the exploratory pilots in the United Kingdom and the validation in Spain. The statistical convergence of usability scores (SUS) at both sites, with means situated in the upper percentile (78.39 and 87.50, respectively), strongly supports the first proposed hypothesis (H1) and suggests that the proposed design is robust against architectural and linguistic variations. A remarkable aspect of these results is the high score obtained in the learnability subscale despite the digital divide in older age; the findings indicate that a well-calibrated multimodal interface reduces the barrier to entry, enabling users without previous experience in Augmented Reality to understand and accept the system. When examining the multimodal interaction in depth, a functional hierarchy is observed in the perception of stimuli where auditory guidance acts as the primary modality. The strong correlation found between voice clarity and overall usability is consistent with our second hypothesis (H2) and suggests that, for this demographic group, verbal instructions mitigate navigation anxiety, acting as a safety net whilst they process visual information. On the other hand, the evolution of the AR arrow’s evaluation, which shifted from a polarised distribution in the initial prototype tests to high and more uniform acceptance in the usability tests with users from the target group, underscores the importance of signal stability. The usefulness of AR cues observed in our study is consistent with recent findings, where AR-based navigation proved significantly more effective for users unfamiliar with the environment [3]. From the perspective of human factors and ergonomics, one of the most interesting findings was the majority preference for hand-held interaction over the hands-free alternative, a behaviour that contradicts certain theoretical assumptions about design for ageing. The qualitative analysis revealed that this choice did not respond to physical limitations, but rather to a phenomenon of transfer of prior technological habits: those participants with greater frequency of smartphone use replicated their pre-existing motor patterns out of habit. This suggests that attempting to impose hands-free solutions, under the premise of facilitating the task, may be counterproductive if it conflicts with the user’s mental models and muscle memory. Keeping the device in hand grants the user a greater sense of agency and control, allowing them to actively manage when to consult the screen and when to attend to the environment. Finally, in addition to performance metrics, the system’s impact on quality of life is reflected in the high ratings of perceived safety and independence support, supporting the third hypothesis (H3) regarding the psychosocial impact of multimodal guidance. Although the sample size invites prudence in generalising the data, the internal consistency of the scale supports the conclusion that AR-assisted navigation, when it respects the user’s inherent habits and guarantees sensory stability, is a viable solution for fostering autonomy in complex environments. A further limitation concerns indoor environments in which visual evidence becomes less distinctive or less reliable, such as crowded and highly dynamic spaces or buildings with repetitive layouts across floors. In the first case, temporary occlusions caused by passers-by or movable objects may reduce the reliability of landmark verification. In the second, visually similar corridors and nearby landmarks may introduce floor-to-floor ambiguity. Although the proposed system mitigates these risks through temporal voting across consecutive frames, partial landmark validation, hierarchical recovery, floor-aware node encoding, and context-constrained visual verification, these conditions were not analysed in isolation in the present study. Since this work was conceived as an initial feasibility and user-acceptance evaluation with the target population, a more specific assessment under crowded, highly dynamic, and strongly repetitive multi-floor conditions should be addressed in future work. Moreover changes in lighting conditions may affect the visual appearance of semantic landmarks and therefore influence the robustness of landmark verification. The present study did not analyse illumination as an isolated variable; however, the system was tested under naturally varying real-use conditions. A dedicated quantitative evaluation under systematically varied lighting conditions remains part of future work. Finally, future iterations of the system will explore an instrumented technical benchmark reporting node-level localisation accuracy, transition-detection latency, rerouting frequency, and, when continuous ground truth is available, metric localisation error. Moreover, future work will include the integration of machine learning algorithms to personalise the interface in real time, adapting the pace of instructions and the size of AR elements to each user’s specific walking speed and visual acuity.

## 7. Conclusions

This study presents and validates an indoor navigation architecture assisted by Augmented Reality and voice, demonstrating that the proposed solution is technically feasible and useful for the older adult population. After analysing the results, it is concluded that the design based on multimodal interaction has achieved high levels of usability and acceptance in the different testing contexts, overcoming the usual technological barriers to entry in this demographic group.

Furthermore, the consistency of the metrics obtained supports the hypothesis that the combination of clear auditory instructions with contextual visual cues acts as an effective compensatory mechanism in the face of spatial orientation difficulties. These indications highlight the evidence that the system not only fulfils its guidance function, but also, through its interface design, fosters a greater sense of control in the user. This capacity to adapt to interaction preferences facilitates a more natural adoption of the technology, reducing the anxiety associated with navigation in unfamiliar places.

Finally, the validated system transcends the utility of transport from one point to another and acts as a tool that significantly increases the perception of safety and autonomy. These factors help to promote active and independent ageing, enabling users to navigate with confidence in complex architectural environments.

## Figures and Tables

**Table 1:** Comparison of representative indoor navigation approach families and Vertex.

Approach Family	Dedicated Infrastructure	Prior Model/3D Map Required	Physical Markers	Visual Processing
Infrastructure-based positioning (e.g., Wi-Fi, radio beacons)	Yes	No/Partial	No	None/Low
Visual tracking and dense 3D mapping	No	Often	No	Intensive
Image-based localisation	No	Yes (image database)	No	Medium
Marker-based methods	No	No	Yes	Low
Semantic graph/Digital building models	No	Yes (digital twin)	No	Low
Vertex (proposed)	No	No (video walkthrough only)	No	Medium (Vision-Language Models)

**Table 2:** Model Specifications and Configuration.

Module	Model/Engine	Configuration	Function
Vision (VLM)	GPT-4o (OpenAI)	Temp: 0.0 (Deterministic) Max Tokens: 60	Semantic verification and contextual QA
Wake-Word	Vosk (via Recognissimo)	Model: ‘small-es’ (Local) Trigger: “Vertex”	Offline voice activation (Edge computing)
Voice (STT)	Faster-Whisper	Model: ‘small’ Precision: float16 (CUDA)	Transcription of complex commands
Voice (TTS)	Android Native	Engine: Google Speech Language: es-ES	Low-latency auditory feedback

**Table 3:** System Usability Scale (SUS) scores by phase.

Phase	*n*	Mean	SD	95% CI
Technical pilot (United Kingdom)	13	78.39	17.34	[67.91, 88.87]
Validation (Spain)	21	87.50	17.57	[79.50, 95.50]

Note: SD denotes standard deviation and CI denotes the 95% confidence interval of the mean, calculated as $\mathrm{CI}=\bar{x}\pm t_{0.975,n-1}\frac{\mathrm{SD}}{\sqrt{n}}$ using the number of valid responses in each phase.

**Table 4:** Rating of specific functions (Scale 1–5). Mean and (in parentheses) Top-box percentage (4–5).

Function	United Kingdom (Pilot)	Spain (Valid.)
Route selection	4.33 (93.3%)	4.42 (91.7%)
Voice clarity	4.00 (66.7%)	4.58 (83.3%)
Map usefulness	3.53 (46.7%)	4.25 (66.7%)
AR arrow usefulness	3.40 (46.7%)	4.25 (83.3%)
Voice interaction	4.00 (66.7%)	3.92 (75.0%)

## Data Availability

The data presented in this study are not publicly available due to privacy and ethical restrictions involving human participants. Source code is available upon request.
